# Biomechanical Texture Coding in Rat Whiskers

**DOI:** 10.1038/s41598-018-29225-9

**Published:** 2018-07-24

**Authors:** Maysam Oladazimi, Wieland Brendel, Cornelius Schwarz

**Affiliations:** 1 Systems Neurophysiology, Werner Reichardt Centre for Integrative Neuroscience, Eberhard Karls University of Tübingen, Tübingen, Germany; 2 Computational Neuroscience, Werner Reichardt Centre for Integrative Neuroscience, Eberhard Karls University of Tübingen, Tübingen, Germany; 3 Department of Cognitive Neurology, Hertie Institute for Clinical Brain Research, Eberhard Karls University of Tübingen, Tübingen, Germany

## Abstract

Classically, texture discrimination has been thought to be based on ‘global’ codes, i.e. frequency (signal analysis based on Fourier analysis) or intensity (signal analysis based on averaging), which both rely on integration of the vibrotactile signal across time and/or space. Recently, a novel ‘local’ coding scheme based on the waveform of frictional movements, discrete short lasting kinematic events (i.e. stick-slip movements called slips) has been formulated. We performed biomechanical measurements of relative movements of a rat vibrissa across sandpapers of different roughness. We find that the classic global codes convey some information about texture identity, but are consistently outperformed by the slip-based local code. Moreover, the slip code also surpasses the global ones in coding for active scanning parameters. This is remarkable as it suggests that the slip code would explicitly allow the whisking rat to optimize perception by selecting goal-specific scanning strategies.

## Introduction

Sensory processing leads to a representation of a sensory object in terms of spatio-temporal brain activity. But how does sensory information reach the nervous system in the first place? The transfer function which projects an object’s relevant characteristics to activity of sensory receptors is specific for each sensory modality. It is dependent on the physics (and/or chemistry) governing the conveyance of object properties to the neuronal receptor. For the fine epicritic tactile sense, the subject of the present work, these processes critically include the frictional system set up by the conjunction and relative movement of sensor and object. Frictional movement is sure to impose a complex transformation of sensory signals by biomechanically converting 3D texture surfaces into spatiotemporal vibrations of the integument – way before the first action potential in a neuron is generated^1^. In this report we focus on rats’ vibrissa, sensor hairs that the animals actively move across textures. We aim at characterizing the transformation of texture identity into vibrissa vibration and compare possible encoding symbols used for this purpose.

Classically two coding symbols have been discussed for fine epicritic touch, ‘intensity’ and ‘frequency’^2^. These two candidate symbols have in common that they are the result of temporal (or spatial) integration of the vibrotactile signal within relatively extended ranges, and therefore have been subsumed under the heading ‘intensive’ or ‘global’ coding strategies^1,3^. Frequency is the outcome of Fourier analysis (e.g. best frequency) while intensity is the outcome of signal averaging (e.g. mean speed). The observation that friction leads to quasi-stochastic short-lived events called stick-slip movements of whiskers (called ‘slips’ here) opened the possibility that slips act as a novel coding symbol^4–6^. Coding in slips differs distinctly from intensive coding strategies by its ‘local’ character in space-time. Thus, extensive integration in space-time is neither needed nor is it appropriate to read out texture information from slips^1^.

Originally, the idea of global coding has been developed without knowledge of slips. Theoretically, in the absence of frictional movements and slips, global coding could directly transform the texture’s 2D profile along the ‘path’ of the vibrissa, e.g. leading to a measurement akin to a contactless distance meter. In reality, however, the conical form^7–9^, and the pronounced elasticity of whiskers^10,11^, inevitably lead to frictional movement and slip generation and renders this simple view unsustainable. Therefore, given the presence of slips, the question arises whether a global code can be considered as optimal to read out texture information contained in slips, or in how far it is able to contribute texture information from other segments of the vibrotactile signal, like oscillations or creepy movements^12–14^.

**Figure 1:**
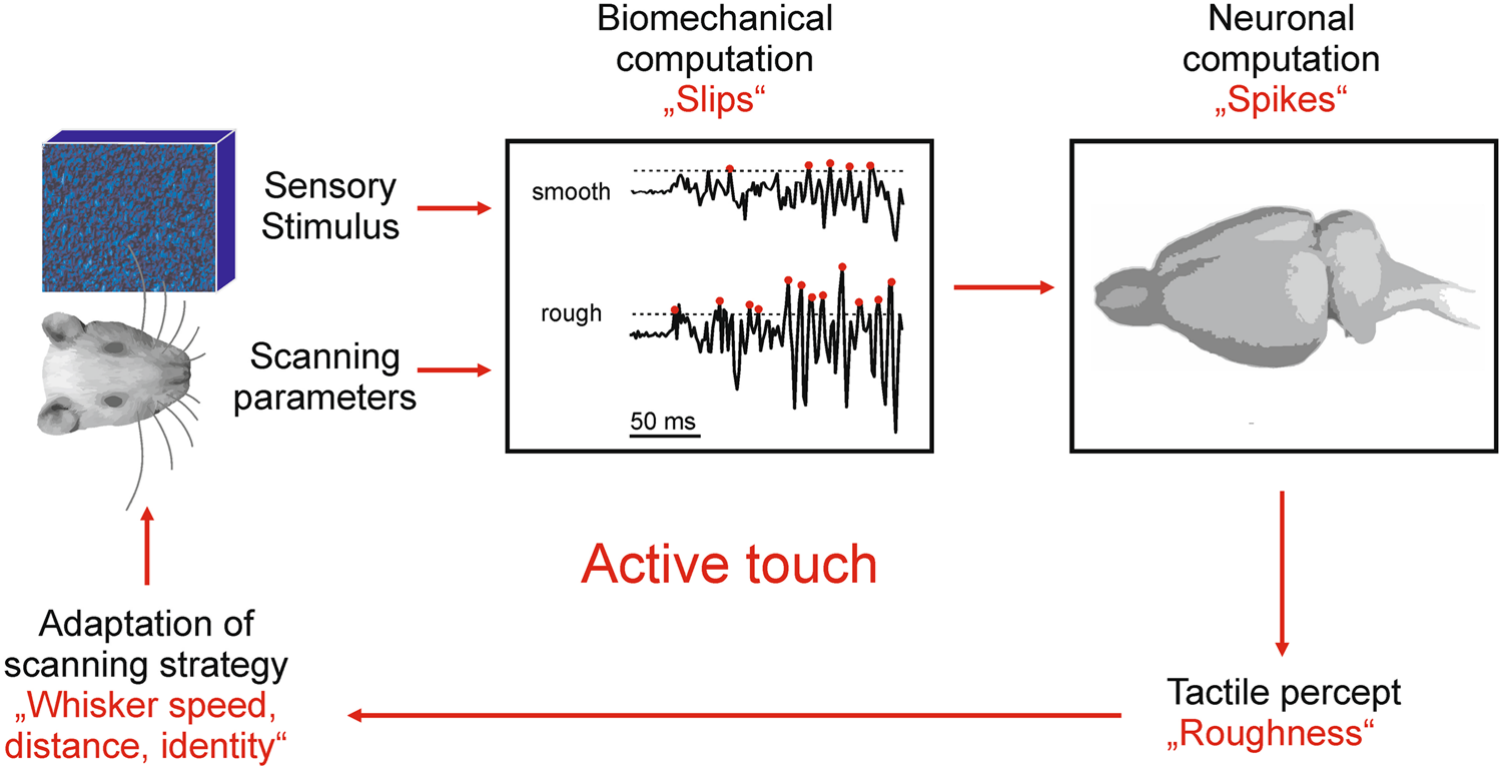
Active touch. The tactile system is an active scanning system. This class of perceptional systems is defined by deployment of energy to the world and sensing the reflections of the deployed energy as a basis for perception. In the case of the whisking rat, the deployed energy consists in kinetic energy, the driving speed with which the whisker is moved across the perceptional target. The sensed reflections of this energy are modulatory movements of the whisker, caused by frictional movement, added to the ego-motion. The modulatory movements are dependent on sensory variables, the texture property, as well as active scanning or contextual variables, amongst them whisker identity, speed, and distance. One instantiation of reflected energy are frictional stick-slip movements (‘slips’) which are encoded into action potentials in the neuronal system (‘spikes’). Whisker vibration and its neuronal representations are therefore the output of a biomechanical computation integrating sensory and contextual variables. This fact allows the brain firstly to read out sensory information, but secondly also allows for a specific adaptation of active scanning strategies to optimize the percept.

A special feature of tactile perception is that it is based on ‘active scanning’, the emission of energy into the world aiming to perceive objects using the physical reflections of these deployments. This feat is shared e.g. with echolocating bats/whales and weakly electric fish, but with no other mammalian sense. By actively whisking against objects, rats choose an active perceptual strategy by selecting several behavioral parameters, amongst them (1) distance of the rat’s head to the object (which determines axial and lateral forces acting on the vibrissa^15^), (2) velocity of whisking, and (3) the identity of the vibrissa(e) used to touch. These parameters of active scanning in turn will determine the frictional movements and thus the probability of occurrence (rate) and the waveform (kinematic parameters) of slips. Besides coding for texture properties, slips will, therefore, inevitably also encode the parameters of the perceptual strategy of the animal (Fig. 1). In this report we will therefore not only assess texture coding using the different hypothetical coding symbols but also determine in how far parameters of active touch modify this code. This knowledge will be decisive to gain insights on what an effective neuronal efference copy^16^ must look like, if it is to effectively separate textural information from the imprints of active scanning.

## Results

**Figure 2:**
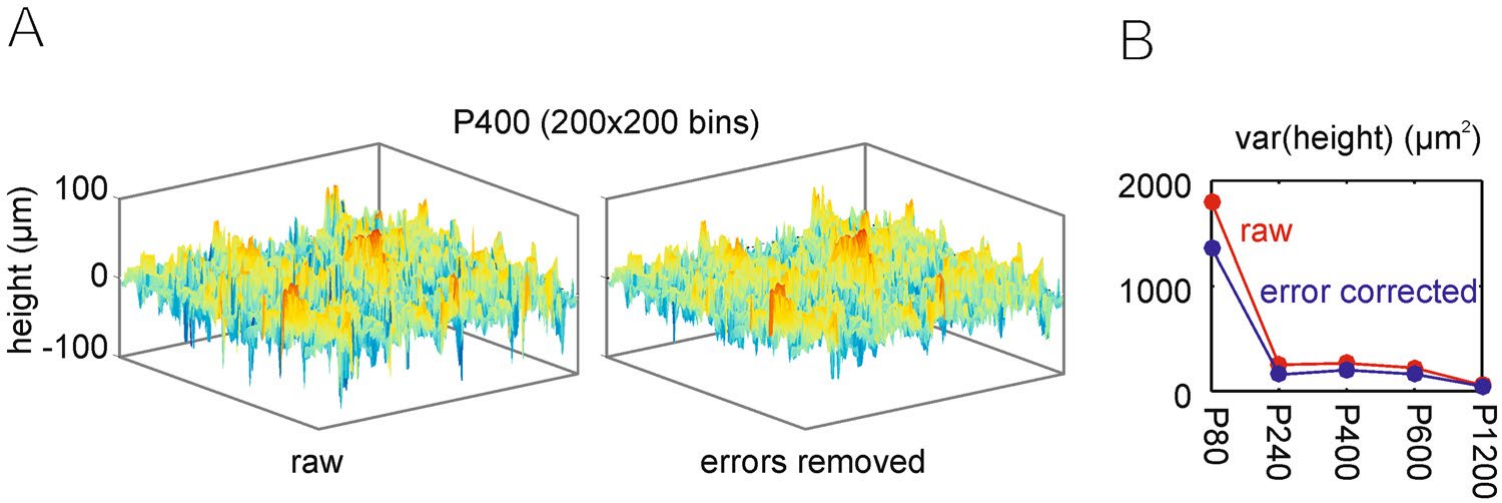
Measurement of sandpaper 3D profile. Sandpapers of different grain size (‘roughness’, cf. Methods section) were used in this study (p80, p240, p400, p600, p1200). Optical micro-profilometry was used to measure the 3D profile of the sandpapers. Profiles contained errors systematically located at the deepest point of the 3D profile (see the dips in negative height in the ‘raw’ measurement). These erroneous dips could be easily demarcated, removed and linearly interpolated. The resulting 3D surface after removal of these errors is shown in the center, and its effect on the variance of the height distribution is shown on the right.

**Figure 3:**
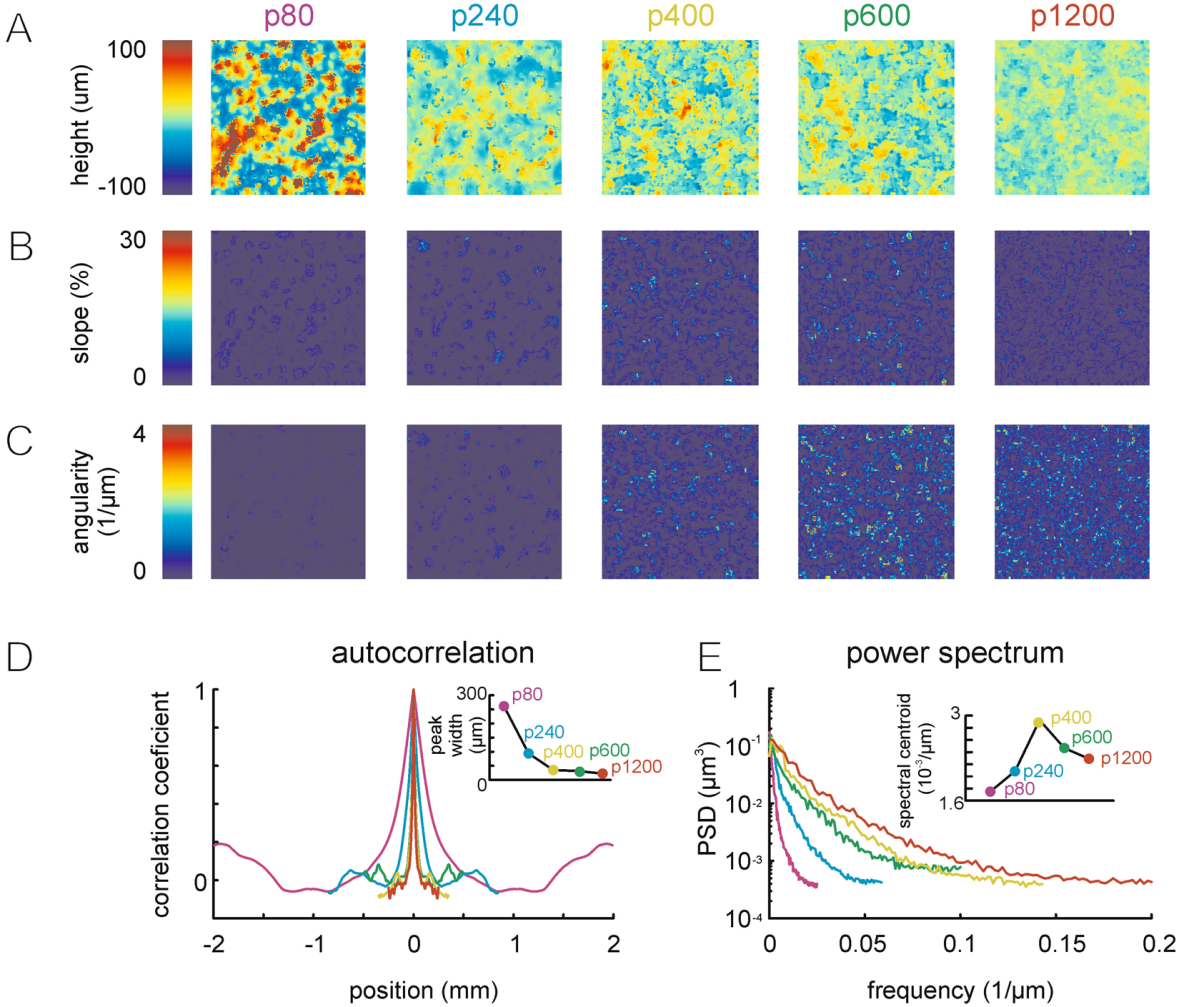
Sandpaper surfaces and characteristic parameter distributions. (**A**) Measurement of the 3D surface of all five sandpapers (color code is height) and the corresponding height distributions. (**B**) Same as in A but the first derivative ‘slope’ is shown. Note that direction of slope is ignored, therefore slope is given in absolute values. (**C**) Same as A and B, but angularity, the second derivative of height is shown. Again direction is ignored and angularity is given in absolute values. (**D**) From the grain sizes used to fabricate the sandpapers a monotonic correlation length of height was predicted. We confirmed this expectation by measuring the width at half peak of the central peak of the spatial autocorrelograms calculated from the 3D height profiles. (**E**) Spatial spectra of the sandpapers. Note that neither the curves nor the spectral centroid reflects the monotonic increment of sandpaper grain sizes.

The textures used in this study were sandpapers with descending grain size from p80, the roughest, via p240, p400, p600 to p1200, the smoothest (see methods for details). Sandpapers of this roughness have been used in various previous biomechanical and encoding studies^4,17,18^, and the range of the resulting vibrotactile signals is behaviorally relevant^19,20^. To quantify the 3D profile of the sandpapers, we measured their height profile using optical micro-profilometer (Fig. 2). From this profile we used the first and second spatial derivative to assess the slope and angularity profile. This was done ignoring the direction of slopes and angularity, i.e. yielding absolute units (percent for slope, and 1/μm for angularity; Fig. 3A–C). We found that larger grain size expectedly goes along with greater length of spatial correlation, a parameter estimated from the width at half height of the central peaks in spatial auto-correlograms (Fig. 3D). The spatial power spectra and their centroids, in contrast, did not show monotonic relationship with grain size (Fig. 3E).

**Figure 4:**
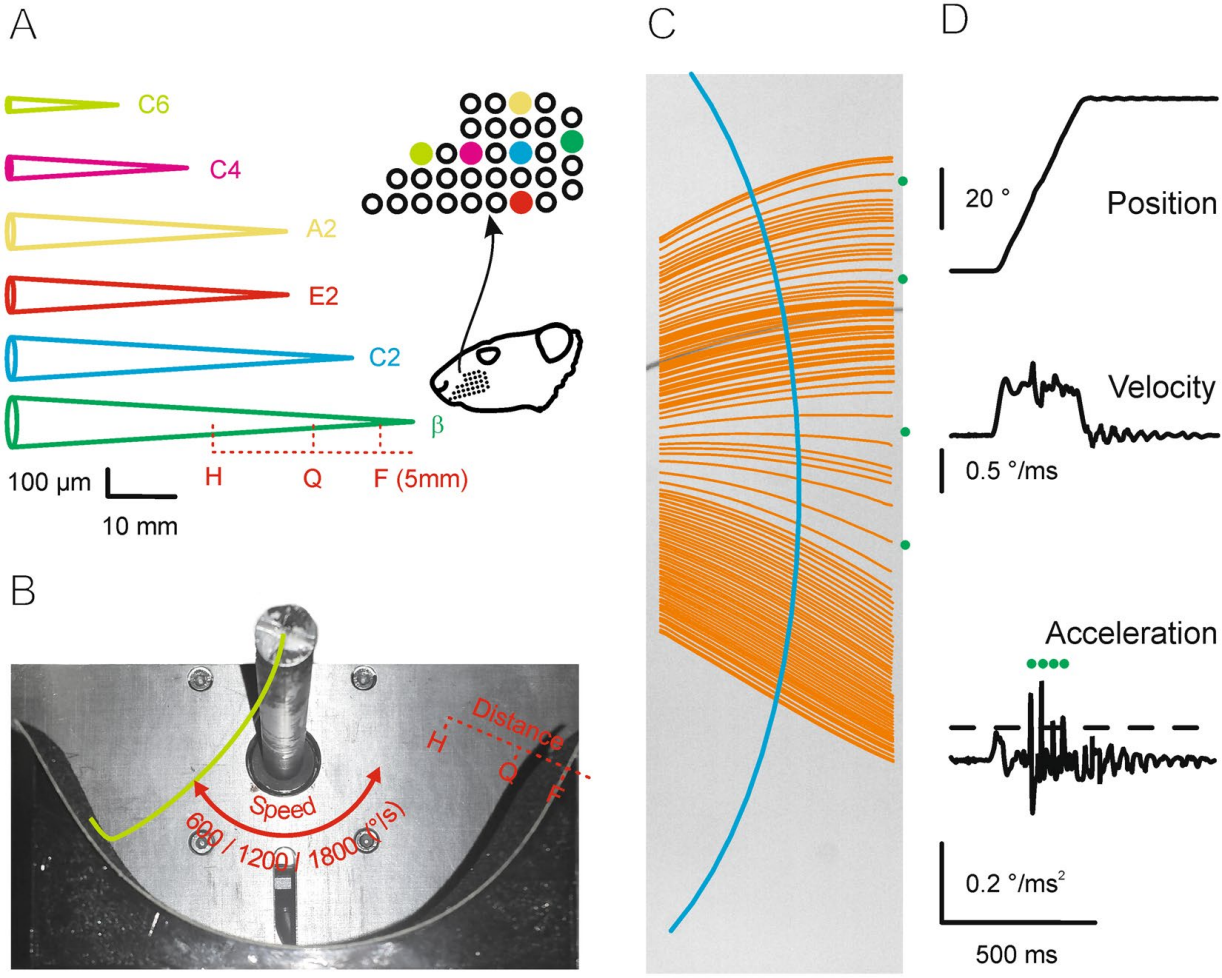
Measurement of frictional movement. (**A**) Selection of whiskers (names and position on the snout) used for this study. The length and diameters were measured and are depicted as schematized cones on the left (for graphical purposes diameters and length use different scale units; intrinsic bending of the whiskers is ignored). (**B**) Frictional movement. The rod (top) was rotated by a stepper motor. The whisker (green) was clamped with the follicular end on the rotation axis to the rod and moved at different speeds and distances against the sandpaper carried by the semicircular shield. For the movement of each whisker three shields with diameters adapted to H (half length), Q (three quarter length) and F (total length minus 5 mm) were used (F was not used with whiskers C4 and C6; Q was not used with C6). (**C**) Example videographic trace of whisker movement. The orange lines depict whisker positions in each frame (4000/s). The blue line indicates the position on the shaft used to extract kinematic traces used in this study (cf. D). Green dots mark the sites at which the whisker acceleration exceeded 2*SD of acceleration measured with movement in air (slips). (**D**) Position, velocity and acceleration traces extracted from one movement cycle (one protraction). The green dots mark the sites in which the whisker exceeded 2*SD of the acceleration distribution observed with movement in air (using the same driving speed). These events were used to extract slip-related kinematic parameters. As in the majority of retraction movements rotational movements occurred, this study analyzed exclusively protraction movements (cutting out the first 15 ms after movement start).

We used 6 whiskers, harvested from one and the same animal, to sweep them across the textures using constant distance (rotatory movement across the textures covering the inside of a cylindrical plane (Fig. 4A,B). The movement of the hair was videotaped at a frame rate of 4000 fps, and the movement of one point of the hair (distance to the follicle 10 mm) was recorded (Fig. 4C,D). In addition to the 6 whisker identities, we tested two further contextual variables: driving speed (600, 1200, 1800°/s), and distance (distance of pivot point to sandpaper H: 50% of whisker length; Q: 75% of whisker length; F: length of whisker minus 5 mm) (Fig. 4A,B). The used rotation speeds were selected to cover whisking velocities actually generated by rats^21^. We noted that retraction movements were often corrupted by turning the hair, i.e. complex rotatory movements at the extreme protracted position. This was not the case for protraction, where only small turning artifacts were observed. In this study we therefore focused our analysis on protraction movement after cutting out possible artifacts at the start of protraction (first 15 ms).

Confirming earlier work^5,6^, the movement was characterized by irregular, jerky movement events, best visible in the velocity and acceleration traces (Fig. 4D). The movements measured 1 mm from the pivot point did not display absolute stick phases (i.e. zero velocity, Fig. 4D), which is expected given the high elasticity of the whisker (points on the shaft may move forward even in case of total arrest of the whisker tip). As the whisker tip was not accessible in our videographic data, we resorted to a pragmatic definition of stick-slip events (which we call slips here): We defined slip events as short events exceeding 2*SD of whisker acceleration as measured in air (a condition of no contact, and thus no slip) (Fig. 4D, acceleration trace). From the resulting slips we measured the peak slip velocity (*sv*) and acceleration (*sa*) and their rate (*sr*). Further, intensity (*i*) was calculated as mean speed and frequency (*f*) as the frequency carrying maximum spectral power.

The definition of these parameters was chosen such that they would represent either local or global aspects of whisker vibration in the time domain. The local nature of *sa* is given by the fact, that it can be instantly assessed from the vibration trace, without further integration or delay. Intensity, on the other hand, is global as it requires the integration (averaging) of the vibration trace within a large time interval. The definition of parameter *f* needs more clarification, as it has been used in different contexts using different strategies: We defined the term as the frequency that maximized the power spectrum (‘best frequency’). Defined as such, *f* is a truly global variable, as it must be extracted from an extended time interval. Our definition of frequency as ‘best frequency’ is motivated by earlier work^17^, and needs to be strictly separated from encoding strategies using richer signal aspects. For instance, the incorporating of more than one spectral element (like e.g. ref.^22^, a study which used the full power spectrum) into the encoding variable would surely reflect global as well as local signal aspects, see discussion).

**Figure 5:**
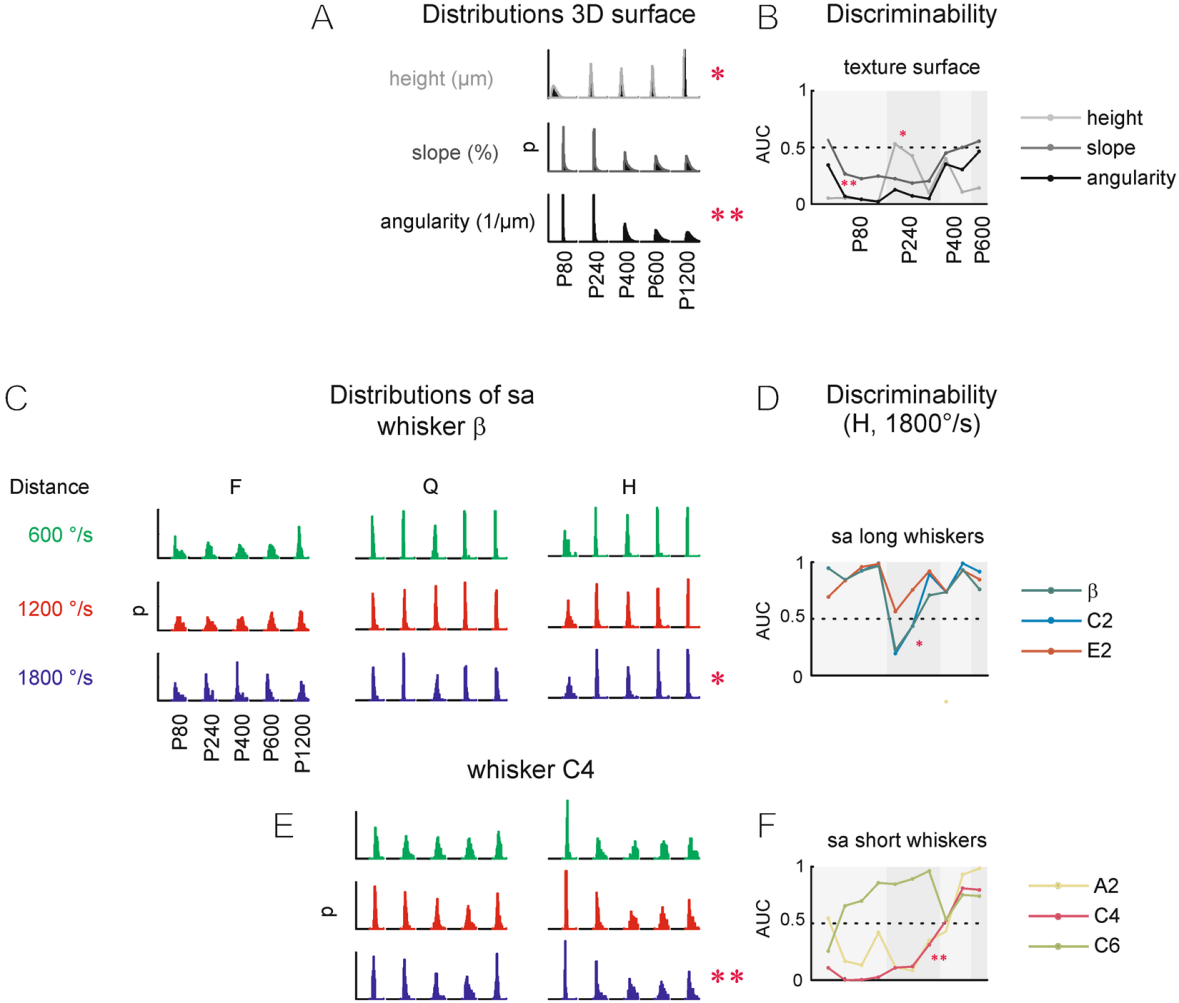
Discriminability of texture and vibrotactile variables. (**A)** Distributions of variables describing the 3D surface of the sandpapers (height, slope, angularity, cf. Fig. 3). The abscissa holds height (range [−200, 200] μm), slope (range [−5, 5] %), and angularity (range [−1, 1]). The ordinate holds probability (*p*, range [0, 0.4]). (**B**) Discriminability of textures based on texture variables [expressed as area under the ROC curve (*AUC*)]. (**C**) Distributions of slip acceleration (*sa*) obtained with different distances (**F**,**Q**,**H**), and whisker velocities (600, 1200, 1800°/mm^2^). The abscissa holds *sa* (range [−5, 5] 1/mm^2^). The ordinate holds probability (p, range [0, 0.4]). Data from whisker β are shown. (**D**) Discriminability of textures based on *sa* for long whiskers. The asterisk marks the correspondence with the set of distributions marked the same in C. (**E**) Same as C but using whisker C4 (note that distance F was not studied with C4 due to its short length). (**F**) Same as D but plotting the shortest three whiskers. The double asterisk marks the correspondence with the set of distributions marked the same in E. The order of sandpapers as marked in A and C (left) is valid for all blocks of distributions shown in A, C and E. The abscissae in B, D and F hold all ten pairwise comparisons of sandpapers ([P80/P240], [P80/P400], [P80/P600], [P80/P1200], [P240/P400], [P240/P600], [P240/P1200], [P400/P600], [P400/P1200], [P600/P1200]). The striped background marks blocks of comparisons starting with p80, p240, p400, and p600.

**Figure 6:**
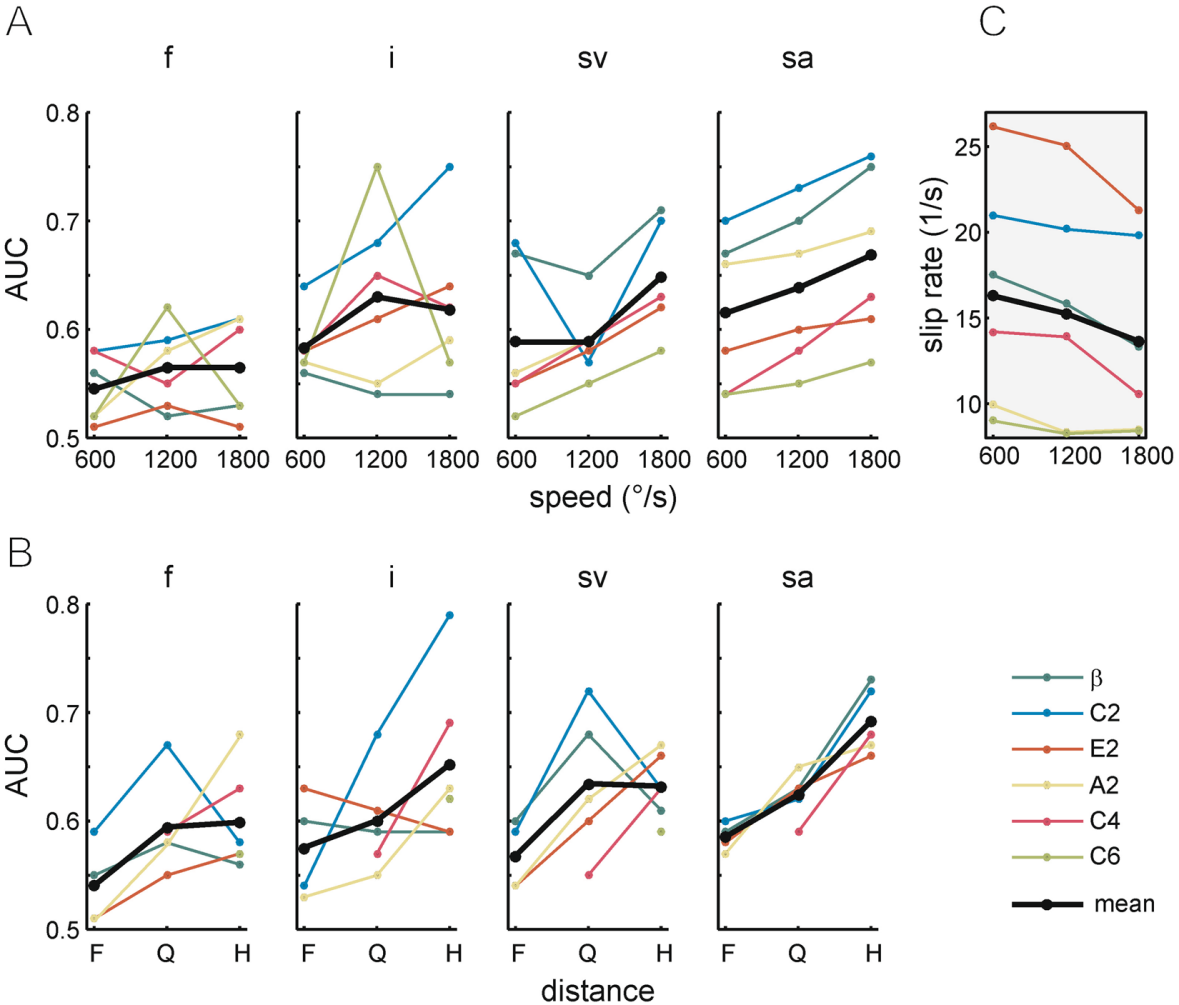
Effect of contextual variables on discriminability. (**A**) Discriminability and speed. The AUC for all neighboring comparisons ([P80, P240], [P240, P400], [P400, P600], [P600, P1200]) for each whisker (color) and averaged across whiskers (black) is shown based on the encoding variables frequency (*f*   ), intensity (*i*), slip velocity (*sv*), and acceleration (*sa*). (**B**) same as A but for contextual variable distance (F: whisker length minus 5 mm; Q: three quarter whisker length; H: half whisker length). (**C**) Slip rate observed with the same speeds as in A. The legend is valid for all panels. Note that all encoding variables in this figure were used at high resolution (thus reflecting the biomechanical facts at the maximal resolution provided by our measurements). Please refer to our considerations that texture information may be carried in a range of signal resolution that cannot be used by rats in Fig. 7.

**Figure 7:**
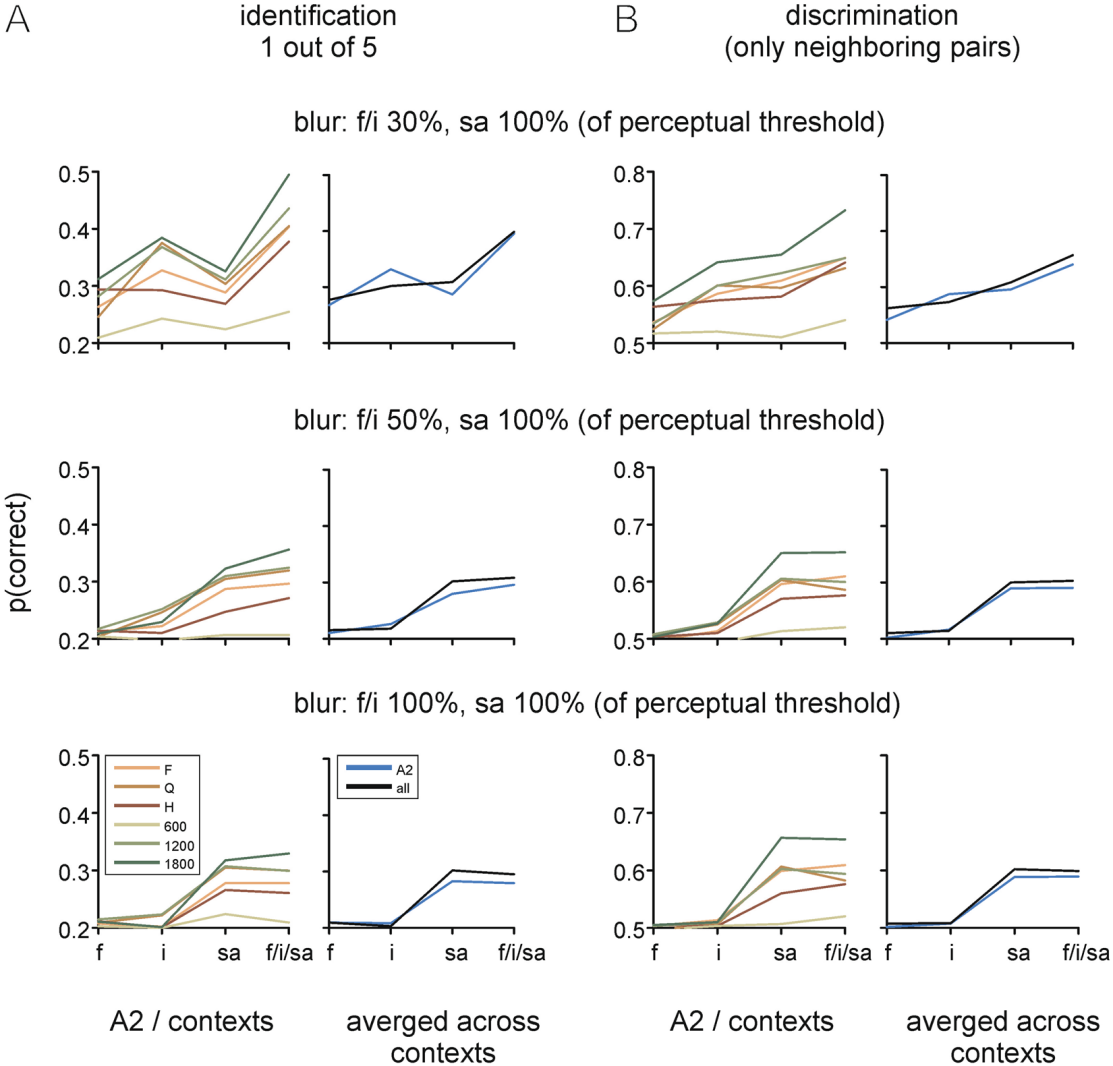
Maximum classification scores obtained from three classifier algorithms using encoding parameters *f* (frequency), *i* (intensity), *sa* (slip acceleration), and *f/i/sa* (all three). (**A**) Performance of identifying a texture (out of 5; random performance is *p(correct)* = 0.2). (**B**) Average performance discriminating pairs of textures with neighboring grain size ([P80, P240], [P240, P400], [P400, P600], [P600, P1200]; random performance is *p(correct)* = 0.5). In both panels the left column holds data from whisker A2: red colors depict the performance for different distances averaged across speeds; green colors depict the performance using different whisker speeds averaged across distances. The right column plots averages across all contexts (for whisker A2 and the total sample of whiskers). The rows depict scores obtained with blurring the input data with respect to perceptual thresholds (100%: setting the known change perception threshold as the standard deviation of the Gaussian used to blur the data; 50%, 30%: setting the standard deviation to 0.5 times the threshold or 0.3 times the threshold; see methods for details). Note that in the top and center rows only the parameters *f* and *i* received reduction of blurring. In these rows, *f* and *i* are thus favored over parameter *sa*, which was always blurred at 100%. All plots contain the entry *f/i/sa* indicating the combined usage of all three parameters as input to the classifiers.

We report biomechanical discriminability of sandpapers based on the highest resolution afforded by our data (Figs 5 and 6). Later, we will consider the known perceptual thresholds of rats, and will ask how much of that biomechanical discriminability the animals might actually be able to access (Fig. 7). We start with asking how a local encoding variable, *sa*, is related to texture variables (height, slope, angularity), and whether there is a systematic dependence on the contextual parameters (driving speed, distance and whisker identity). Texture variables expectedly reflected sandpaper identity, however, there was no consistent relationship across the variables to grain size (Fig. 5A). Height was distributed more widely with larger grain size, while slope and angularity distributions showed quite the opposite - a monotonic decrement of range with larger grain size. Pairwise discriminability, therefore, differs with the three texture variables. Figure 5B plots the discriminability obtained with all pairs of sandpapers (starting with all combinations of P80, then P240 and so forth). While height well separates those pairs containing extreme grain sizes (P80 and P1200), it only provides poor discriminant power in the mid ranges of pairs. Slope and angularity show roughly the opposite. They were very good criteria to separate mid-range pairs but lacked discriminability with pairs in the extreme ranges of grain sizes (pairings of P80 and P1200). In summary, discrimination based on texture variables appears more complex than expected, but overall, appears to provide very good discriminability.

The next question then is whether the discriminability afforded by sandpaper surfaces is reflected by whisker vibrations. In the exemplary case of whisker β (shown in Fig. 5C), the distributions of *sa* obtained with H and 1800°/s and height were of similar shape, and negative height was related to high *sa* (compare the distributions marked with single asterisks). In pairwise texture comparisons of the three largest whiskers (β, C2, E2), we therefore found similarities in discriminability of height and *sa* (ignoring the sign change due to the mentioned inverse relations): Very good discriminability is provided in the comparisons including the extreme grain sizes, but a lesser one in the mid-range of comparisons (Fig. 5D). Interestingly, smaller whiskers (A2, C4, C6), while still holding considerable discriminant power, show qualitatively different behavior (Fig. 5E): Their distributions revealed some relation to slope and angularity distributions (cf. distributions marked with double asterisk), translating into discriminability curves across pairwise comparisons that largely mirrored the ones of slope and angularity (Fig. 5F). A peculiar characteristic of the smallest whisker (C6) was that, while showing best mid-roughness discrimination like the other small ones, it did so using a sign change of discriminability (AUC > 0.5 instead of AUC < 0.5). In summary, we conclude from these results that slip accelerations contain substantial texture information. We observed, however, a non-monotonic, highly complex arrangement of coding of texture variables across whiskers and sandpaper grain sizes. The largest whiskers may be able to pick up discriminant properties of height distributions, while smaller whiskers may focus more on those provided by the higher derivatives slope and angularity. Further, depending on whisker type and/or range of grain size, textural differences can be encoded by increased or decreased slip accelerations.

Next, we aimed to more systematically portray the roles of the contextual variables ‘whisker identity’, ‘driving speed’ and ‘distance’ for each of the coding symbols (*f*, *i*, *sa*, and slip velocity [*sv*]). To this end we present discriminability (*AUC*) separated for these seven variables (Fig. 6), averaged across comparisons of sandpapers with neighboring grain sizes ([P80, P240], [P240, P400], [P240, P600], [P400, P600], and [P600, P1200]). First of all, discriminability was highest in the slip-based encoding variables with slip acceleration providing superior discriminability (rank order *sa*, *sv*, *i*, and *f*). Further, discriminability was also dependent on all three contextual variables (combined with all encoding variables) to varying degrees: Discriminability tended to be higher with longer whiskers, but rank orders did not exactly match those of length. Similarly, AUC tended to order monotonically with increasing speed and decreasing distance. Overall, with slip acceleration these relationships were more consistent and monotonic than with any other variable. In summary, besides providing highest discriminability, slip acceleration also provides superior consistency when contextual variables like driving speed and distance are varied. Interestingly, while discriminability based on slip acceleration rose with speed, slip rate consistently decreased on average by 2,65/s (Fig. 6C). This reveals that the better discriminability provided by individual waveform is countered by less waveforms available per protraction sweep.

Finally we strived to quantify the relative information that global and local variables carry about texture identity using more complex and non-linear classification schemes. To this end we employed two support vector machine algorithms with linear and radial basis functions as well as logistic regression (Fig. 7), to separate the textures based on the observed biomechanical whisker parameters. We trained the classifiers to the three single candidate variables frequency, intensity, and slip acceleration, and performed a 10-fold stratified cross-validation on the training-set to tune the hyper-parameters for each classifier and selected the classifier with the best cross-validation score. The final score was found by determining the median top accuracy of the best classifier on the validation set, i.e. the average probability by which the classifier correctly (*i*) identifies either the texture identity (amongst the 5 possible sandpapers)(Fig. 7A), or (*ii*) the correct texture when presented pairwise with neighboring grain sizes ([P80, P240], [P240, P400], [P240, P600], [P400, P600], and [P600, P1200]; Fig. 7B). To allow a first access to potential effects of the animals perceptual limits we repeated this analysis with step-wise reduction of the resolution of input variables. We deem this important, as e.g. the resolution of best frequency used so far was 1 Hz, a frequency difference that, for all we know at present, is far from being perceptible by rats. It follows that discriminability carried by frequency differences near the resolution of the data is likely going to be irrelevant for perceptual purposes. Psychometric data on whisker-based perception specific for our discriminant variables is still scant. We orient ourselves on measured change thresholds of frequency, intensity and slip acceleration (see details in methods) using pulsatile stimuli with starting values well within the parametric range measured here^23–25^. These change thresholds have been measured only from one starting point, and are likely to vary across the behaviorally relevant parametric range. Nevertheless, we hold that they - at the very least - should provide a good starting point to qualify biomechanical discriminability with behavioral constraints. In face of this uncertainty we followed a strategy of graded decrement of resolution. We started to blur the input data with a Gaussian with an SD equal to the known change threshold (and call this 100%). Obviously, this filter should largely eliminate texture information contained in the subthreshold ranges of the rats suggested by the cited studies. We then repeat the analysis with 50% and 30% filters (SD of Gaussian 0.3 or 0.5 times the respective psychometric threshold) to provide an intuition about the effect of information if thresholds were lower than reported so far. Further, as we aim here to demonstrate the suitability of slip acceleration for tactile discrimination, we decided to give this parameter the most unfavorable conditions (i.e. always reducing resolution with the 100% filter width). If, under these conditions, we find that slip acceleration provides superior discriminability we will have made the best possible case in favor of it. Using this strategy we are able to demonstrate the following facts (Fig. 7): Firstly, *sa* filtered at 100% outperforms *f* and *i* based discriminability averaged across the six whiskers. This is true with all blurring schemes including giving *f* and *i* a three-fold better chance (i.e. reducing the filter to 30%). Secondly, discriminability provided by *f* and *i*, if present (top row of plots in Fig. 7), is non-redundant to information contained in *sa*: If the classifier is fed with all three variables it performs better than with any of those alone. Thirdly, the discriminability conveyed by *f* and *i* variables decreases consistently and is close to random performance when data resolution is decreased to match the perceptual change threshold (top to bottom row of plots in Fig. 7; note random performance is 0.2 for the identity task and 0.5 for pairwise discrimination). Fourthly, this assessment largely holds with a single as well as combining all whiskers. Finally, confirming the previous analyses, contextual variables (velocity and distance) thoroughly modify discriminability. As seen in the previous section, high whisker velocity, by and large, is better than low whisker velocity. An interesting difference, however, is notable with whisker distance: with the AUC classifier, H was observed the best discriminant variable, while the more versatile classifiers used here were found to better extract information contained in slips with longer whisker distances (Q, F).

## Discussion

We compared biomechanical variables of vibrissa movement, while in dynamic contact with textures, with respect to their ability to provide the basis for texture discrimination. Particularly we pit local, (near-) instantaneous kinematic variables extracted from short slip events against the classical global, intensive parameters frequency and intensity. We found that all parameters contain substantial texture information useful for discrimination. However, local slip variables, in particular slip acceleration, turned out to be superior for texture discrimination, both in terms of the strength of discriminability they supported and their consistent behavior in face of varying contextual variables like driving speed and distance of the follicular site of the whisker to the texture. We further find that the suitability of the classic variables frequency and intensity for texture discrimination is seriously hampered when considering known perceptual limits of rats, while the presently known rats’ perceptual ability seems to be more in tune with texture information conveyed by slip accelerations.

We used sandpapers to test these properties, as has been done by many previous studies^4–6,17,26^. This makes our result comparable to that literature, but at the same time restricts the observations to a limited set of real textures. Whether and how vibrations measured on sandpaper generalize to a wider array of surfaces must be tested in future studies. Sandpapers are usually sorted according to roughness, a technical category of abrasiveness. Roughness is also one of the most robust perceptual dimensions used by humans to describe tactile experiences. Psychophysical work has often emphasized that the most important feature giving rise to human roughness perception is thought to be the size of elemental features (wavelength) constituting a tactile texture^27–30^. This has also been borne out from experiments evaluating human similarity judgements across vast arrays of varying textures. Roughness was consistently found to be amongst 2 or 3 independent tactile dimensions^30–32^. Expectedly our detailed measurements of the sandpaper surfaces confirmed that increasing grain size (i.e. roughness) is accompanied by larger wavelength, as estimated by correlation length (Fig. 3C). However, height distributions of sandpapers and their derivatives (slope and angularity) did not reflect this monotonic relationship in a consistent and simple way (Fig. 5B). One possible reason for this discrepancy is that the sandpapers used here are surfaces tightly packed with grains, such that height profiles are not exclusively determined by grain size alone (Fig. 3A). Moreover, single grains, as they appear in profilometry images, are corrugated and vary in form and size. These properties likely compart particular richness to the surface’s frequency spectrum - particularly at higher frequencies. We therefore do not expect that local features (single grain size and surface) overly determine global features (as would be expected e.g. from identical spherical grains sprinkled sparsely on a flat surface).

Confirming this expectation, we found that different surface variables show different and highly complex relationships to texture identity (Fig. 5A,B). This fact questions the existence of a simple relationship of roughness with spatial surface variables on one hand and biomechanical variables of whisker movement on the other. In line with these doubts, our data show a highly complex reflection of surface properties by variables describing whisker movement, which seems to be affected by the texture but also by contextual variables velocity, distance and whisker identity (Figs 5C–F, 6 and 7). These considerations open two important questions that future research needs to address: 1) Is roughness a valid dimension of rats’ whisker based tactile perception? 2) Would, as the dominance of roughness as a perceptual parameter in humans suggests, papillary ridges of the fingertip show frictional movement parameters that are better aligned with roughness?

We wish to stress that from the discriminant analysis performed here we do not claim to have identified measures of optimal discriminability. Rather, we aimed at finding arguments for the relative importance of information carried by global vs. local variables. It is noteworthy that our definition of ‘localizedness’ is in the time domain, as in principle information can be contained in variables defined in time or frequency domains. As we use the terms here ‘frequency’ is global in time but local in frequency, ‘intensity’ is an average across time or frequency, and therefore is global in both, and slip acceleration is local in time and global in frequency. We note that our definition of the ‘frequency’ variable distinctly differs from that used in previous work^22,33^, in which ‘frequency’ was determined by a broad array of spectral elements, and thus, likely incorporated also local features in time. We demonstrate that classifiers fed by a combination of intensity, frequency and slip acceleration cues improve upon the performance shown with each feature alone. This finding corroborates earlier work that reported partly non-redundant information about texture identity in global varibales^17^, and may provide a biomechanical basis for the fact that global variables can support a certain degree of psychometric performance in case local cues are absent^24,25^. However, the observation that most relevant texture information in frequency and intensity variables seems to be conveyed in a parametric range that, at least at the present state of knowledge, may well be imperceptible by rats^23–25^ (Fig. 7), is a reminder that more detailed mapping of the perceptual range is needed before the exact mix of encoding variables used by rats can be determined.

Our quantifications confirm previous suggestions that kinematic profiles of stick-slip events carry texture information^5,6^. Moreover, our findings provide a biomechanical counterpart for prevalent physiological and behavioral findings: Firstly, it is a well corroborated fact, that short kinematic events are readily encoded by neurons on the ascending tactile pathway^4,23,26,34–41^. Secondly, waveforms of short events have been found to be perceptually and behaviorally relevant^23,38,41,42^. We extend the previous insight by showing that local cues provide dominant information about texture identity – surpassing the one provided by global variables. This rank order fits behavioral results obtained from a direct comparison of local vs global variables. Discrimination performance of passive whisker deflections based on local cues by far beats the one reached with pure global cues^23,41^.

In our measurements slip acceleration provided the superior basis for texture identification. This is contrasted somewhat by the tendency of encoding studies to emphasize velocity coding^35,37,42^. However, these reports sometimes specifically included contributions from other kinematic variables, and others have found that acceleration is dominantly encoded^43^. Moreover, cortical spike responses to slips have been shown to be of low probability, but clearly related to slip acceleration^26^. A systems identification study using a broad band stimulus found that several kinematic aspects are linked to different degrees and with different temporal relationships to the encoding spike in primary afferents^40^. In this view, the whole waveform stretching across about 10 ms and several kinematic aspects from position to acceleration (and possibly higher derivatives) may be encoded, such that combination of different aspects and temporal relationships leads to higher information content in the neuronal code. We therefore hypothesize that it is unlikely that rats base their perception on coding of a single variable. The superior information rate of a combination of kinematic variables found in the neuronal code suggests that rats have optimized information extraction by encoding a combination of several kinematic variables.

We varied three contextual parameters that, during the process of tactile perception, are under the control of the animal: whisker identity, driving speed, and distance to texture. Of these variables, whisker identity, surprisingly, was the one mapped in most complex ways on the encoding variables. Notably this was true considering global and local encoding variables (Fig. 6). Interestingly, the longer whiskers may be tuned to different surface parameters as compared to smaller ones (note that all whiskers tested here were obtained from the same animal). This rather complex relationship in the end may introduce only little disadvantage, as active vibrissal scanning will often bring a whole bundle of whiskers or even all of them^44^ in contact with the probed texture. Nevertheless, it will be of importance for further studies to focus on possible behavioral biases to use different whiskers when faced with different tasks^45^. Distance, the second contextual variable, also held considerable inconsistencies in encoding. The type of classifier used (AUC vs. more complex machine learning algorithms) determined whether relevant texture information could be extracted from kinematic traces measured at different distances. Assuming that the rat perceptual mechanisms likely provide complex decoding capabilities, we expect that information encoded with varied whisker distances will be accessible by those animals. Finally, driving speed was the most consistent influencer of discriminability (with global and local encoding variables). Slip acceleration, besides being the variable conferring highest discriminability, was also the most consistent in providing texture discriminability when subjected to varying speed (and distance) contexts. It is worth to note, that if the global variables were a perfect reflection of the 3D geometry of the texture surface, the discriminability conferred by them should be independent from driving velocity. The fact that this is not the case, suggests that global variables do not exclusively measure 3D geometry. Rather, we deem it highly likely that frictional movement (certainly slips, but possibly also other subthreshold whisker vibrations) are contained in the global variables and determine their dependence on context. This is supported by the general match of context influence to that seen with slip-kinematics.

The usage of sandpaper surfaces provided rich and natural-like discriminanda, which clearly demonstrate that contextual influence can affect global as well as local encoding variables and that a dominant contribution may be conveyed by contextually specific frictional movements. It is not clear whether the existence of context specificity of encoding may be boon or bane for texture perception. In any case, contextual specificity provides a purpose for flexibility in movement strategies when solving a task involving active tactile perception^46,47^. The possibility that frictional processes may determine palpation strategies has rarely been studied and must be subject of focused research in the future.

## Methods

### Measurement

Textures consisted in sandpapers of 6 different grit sizes: P80, P240, P400, P600, P1200 (part of the standard series issued by the Federation of European Producers of Abrasives with mean grain diameters of [201, 58.5, 35.0, 25.8, 15.3] μm, respectively). The 3D profiles of these sandpapers were mapped using an optical micro profilometer (MicroProf, FRT, Germany). The resulting profiles (order as above) had a size of 200 × 200 pixels and spanned an area of [4000^2^, 1700^2^, 1000^2^, 700^2^, 500^2^] μm^2^. In the valleys of the 3D profile, the measurement sometimes failed and generated strictly localized errors encompassing a few pixels. As the measuring device set the erroneous measurements to extreme values they could be securely detected and separated from instances where the surface approached extreme height by an additional slope criterion [slope in neighboring pixels above 95% percentile]. The artefactual bins as well as the bins neighboring a detected error bin were removed. The number of pixels thus removed for each height profile (order as above) was [756, 1158, 829, 844, 2048] out of 40000. The removed values were linearly interpolated (Fig. 2).

The height profiles of the sandpapers were differentiated twice to obtain measures of ‘slope’ (1^st^ derivative), and ‘angularity’ (2^nd^ derivative) (Fig. 3A–C, top). For sampling the distributions in panel 5A, the interpolated values (see last paragraph) were omitted. The differentiation was obtained by moving a 2 × 2 bins window across the height profile. Within each window the difference in the two cardinal directions (rows and columns of the profile) were assessed by averaging the two differences in each of the directions. The length of the vector sum of the cardinal difference vectors (along row/column) yielded the slope (and angle) within the 2 × 2 window. Angularity profiles were calculated the same way from slope profiles. The height profiles were used to calculate the spatial power spectrum and the autocorrelograms (Fig. 3D).

A set of rat whiskers A2, C2, E2, C4, C6, and β was plugged from a dead animal (in fact all whiskers studied here stemmed from the same animal). Care was taken that the follicle was included in the plugged whiskers and the hair shaft was devoid of kinks. Length and diameter at the follicle was measured using microscopic pictures. As reported before^7,8^, the whisker were approximately of conical shape. The most salient divergence from the cone shape was that the last few hundreds of microns toward the tip of the whisker, the taper was irregular, and the tip was cut at a width of about 5–10 μm. The [widths at base (μm), width at tip (μm), length(mm)] were: A2: [162.79, 3.07, 41.05], C2 [184.29, 7.42, 49.45], E2: [178.15, 3.59, 41.71], C4 [124.36, 4.61, 26.48], C6 [81.93, 2.05, 17.96] β [190.43, 8.72, 55.90]

The whiskers were clamped with the follicular site to the rotational axis of a rod sticking out perpendicular to it (Fig. 4B). By rotating the rod using a stepper motor (AR66ACD, Orientalmotor, Tokyo, Japan controlled by Built-In Controller Package and data setting software MEXE02 and interfaced via CC05IF-USB to the PC) the whisker was rotated around its base. A high speed camera (MotionBLITZ EoSens® Cube6, Unterschleissheim, Germany) was positioned 50 cm above the rotational plane of the whisker and filmed the movement of the whisker (Tokina 100 mm f/2.8, AT-X PRO – Macro, Kenko Tokina Co.,Ltd., Japan) 160 × 627 pixels, 16 × 16 μm^2^/pixel, 4000 fps). The sandpapers were mounted on circular plastic shields mounted such that its center point coincided with the rotational axis of the whisker motion. Hence the distance of the follicle to texture was constant for the entire rotational movement. The shields were printed using a 3D printer (Ultimaker3, Ultimaker, Netherlands) at diameters adjusted such that the distance of the follicular end to the texture was (F) the length of the whisker minus 5 mm, (Q) 3/4 of the whisker length and (H) 1/2 of the whisker length. We took care that the whiskers intrinsic bending lay in the plane of movement direction. One measurement cycle consisted in forward movement (60°; ‘protraction’) across the texture (Fig. 4). The hair was video-imaged for 100 cycles capturing the hair shaft exposed in free air, ignoring the tip region engaging with the texture.

### Analysis

Whisker trajectory was read out from the videos at a constant distance of 10 mm from the base. Figure 4C shows a series of whisker shapes obtained from a subset of frames obtained with the high speed camera during one movement episode. From the total set of frames the whisker position trajectory was constructed and differentiated twice to obtain position, velocity, and acceleration traces (Fig. 4D). Slips were detected in acceleration traces by applying a criterion, which was determined as 2*SD observed in control measurements when whiskers moved in air (no texture) (broken line in Fig. 4D). For each detected slip event the time of occurrence, peak velocity, and peak acceleration was assessed. In addition, for each trace the power spectrum, and the mean speed was calculated to extract the intensive parameters frequency and intensity. Before calculating the power spectrum using fast fourier transform, the signal was band-pass filtered (cut off frequencies [30, 250] Hz; Chebyshev type II filter of 5^th^ order) and a Hanning window (raised cosine window) was applied.

Discriminability was determined for a pair of textures, calculated as the area under the ROC curve (*AUC*). *AUC* is the probability of correct classification of a binary classifier (using varying thresholds to strip off the observer bias) confronted with a randomly picked whisker trajectory obtained with touch on one of the textures^48^. Therefore *AUC* = 0.5 signifies random performance while *AUC* values of 0 and 1 signify perfect discrimination.

We calculated three versions of slip-based discriminability using distributions of (*i*) peak velocity, (*ii*) peak acceleration, and (*iii*) slip rate. Frequency-based discriminability was based on distributions of best frequencies found by maximizing power spectra, and finally, intensity-based discriminability was calculated as mean speed (after subtraction of the rotational, ‘driving’ speed due to the motor).

Finally, we cross-checked the results of the *AUC* analysis using three different one-against-all classifiers and 10-fold cross-validation: (1) Support Vector Machine (SVM) with linear and (2) Radial Basis Function (RBF) kernels^49^ and (3) Logistic Regression^50^. We used the standardized Scikit-learn implementations (version 0.18.1^51^). For each dataset (referring to the data from one whisker at one distance and one speed) we estimated the optimal hyper-parameters for each classifier in the following way: we first split the data in train and test set (in a stratified fashion), then we perform a grid-search over the hyper-parameters with a 10-fold stratified cross-validation. We select the classifier with the best mean cross-validation score. In Fig. 7 we report scores of the best classifier (according to the cross-validation score on the training set) on the hidden test set.

As texture information is contained in very small differences of frequency and intensity data (Fig. 7) that we deemed clearly below the perceptual threshold of the animals^23–25^, we repeated this analysis using blurred input data to the classifiers. We took estimates for perceptual thresholds of changes in *f* and *i* variables from refs^22,23,40^. These studies used pulsatile discriminanda that were generated from identical pulses (i.e. instantaneous coding was excluded to contribute to the performance). Despite differences in psychophysical design (and stimulus reward contingency), 4 rats (three from ref.^23^ and one from ref.^24^) showed threshold performance with intensity differences of ~450 deg/s and frequency differences of ~36 Hz (cf. experiment 1b and 2b in ref.^23^, see their Table 1 and Figs 2 and 3). As has been reported later^25^, rats likely use intensity rather than frequency to generate their percept. Despite this qualification we deem it appropriate to consider both estimates (for *f* and *i*) as a lower bound to rats’ perceptual threshold of change. Ref.^23^ in addition reports a threshold amplitude change of pulses of 1.14 deg (their experiment 1). This translates into an acceleration change threshold of 23600 deg/s^2^ (cf. Table 1 and Fig. 2 in ref.^23^). To demonstrate the effect of excluding texture information below these perceptual limits we blurred the input data to the classifiers by substituting each data point by a random pick form a Gaussian (with a mean set to the value of the point and standard deviation equaling the respective perceptual threshold). In other runs of the classification procedure we used fractions of this range (50% and 30%, cf. Fig. 7).

### Data availability statement

The datasets generated during and/or analysed during the current study are available from the corresponding author on reasonable request.
